# Targeting inflammatory pathways in hepatocellular carcinoma: recent developments

**DOI:** 10.1007/s12672-025-03035-8

**Published:** 2025-06-22

**Authors:** Fulufhelo Tabitha Ramaite, Sanah Malomile Nkadimeng

**Affiliations:** Department of Life and Consumer Sciences, Collage of Agriculture and Environmental Sciences, University of South Africa, Florida Campus, Roodepoort, Johannesburg, 1709 Gauteng South Africa

**Keywords:** Inflammation, Liver cancer, Tumour microenvironment, Cancer therapies, Cytokines

## Abstract

Chronic inflammation is a well-established driver of malignancy in many various cancer conditions. Hepatocellular carcinoma (HCC) is a significant illustrator of cancer linked to an inflammatory response and known to arises from prolonged liver damage. Inflammation is ranked number five of the most common factors in the occurrence of cancer conditions globally. Furthermore, it is placed third as the leading reason for cancer-related deaths, accompanied by nearly a million new diagnosis and fatalities annually. Pathological inflammation causes an ongoing liver damage and regeneration, which leads to fibrosis, cirrhosis, and eventually HCC. Although various factors contribute to HCC, a common mechanism is the inflammatory response that is triggered by cell death and the resulting inflammatory cascades. This review assesses recent progress in liver cancer research, focusing on how inflammatory pathways contribute to disease progression. It highlights the role of cytokines along with other inflammatory mediators in the progression of HCC stemming from chronic liver damage. The review also explores new therapeutic approaches targeting inflammatory pathways, including novel compounds and synthetic agents, such as IL-6 receptor antagonist and NF-κB pathway blockers and their potential for effectively treating and preventing liver cancer. Furthermore, it addresses current limitations and challenges in targeting inflammatory signalling and outlines future research directions to advance the clinical development of anti-inflammatory agents for liver cancer prevention and treatment.

## Introduction

Inflammation is a natural defensive reaction to damage or illness and in normal circumstances, it involves processes that prevent tissue harm [1]. When inflammation remains unmanaged and prolonged, it leads to undesirable conditions, which are linked to many chronic illnesses such as liver cancer [2]. According to the International Agency for Research on Cancer (IARC) conducted in 2020, liver cancer was the sixth most common type of cancer worldwide in 2020 and placed as the third major cause of deaths related to cancer. Furthermore, IARC estimated more than 900,000 newly documented cases of liver cancer across the globe, which resulted in approximately 830,000 deaths attributed to the disease. As aresult, liver cancer represents an increasing global concern [3] and in Africa, it must be handled effectively to prevent the increased incidence and death rates [4]. Furthermore, it is estimated that the demographic surge and increasing age will result in a 70% rise in new cancer cases by 2030 [5]. Cancer arises from unregulated proliferation of certain bodily cells capable of spreading to different areas, such cells may cluster together to create tumours, which are masses of tissue [6] which can be malignant or benign [7]. Malignant tumours are cancerous, and infiltrate surrounding tissues. Additionally, they can travel to remote areas of the body to develop new tumours through metastasis, whereas benign tumours are not cancerous and do not grow into or invade neighbouring tissues [7].

This review will explore the latest developments in comprehending the physiology along with liver cancer management, accentuating significance of inflammation as a key driver in its progression. As inflammatory processes are increasingly recognized as a central element in the evolution of liver cancer, grasping these processes is fundamental to the formation of specific treatments. Liver cancer, ranked among widely occurring cancers and the third most frequent contributor to deaths related to cancer, underscores the urgent need for novel treatment approaches. This review seeks to deliver a comprehensive examination of these mechanisms and recent therapeutic strategies. Especially looking at recent developments into the impact and role of inflammation and its cascade pathways in the progression of cancer. By examining both the physiological underpinnings and emerging treatments of liver cancer, this review presents important perspectives for researchers and clinicians seeking to enhance therapeutic strategies.

## Methodology

This literature review was undertaken to examine recent advancements in the comprehension of liver cancer physiology, specifically emphasizing the influence of inflammation on the progression and treatment of hepatocellular carcinoma (HCC). We used information that was sourced from publications released between 2003 to 2024. The following research electronic databases were used: Research Gate, PubMed, ScienceDirect, and Google Scholar. The search utilized a combination of keywords, including “hepatocellular carcinoma,” “liver cancer,” “inflammation,” “cytokines,” “TNF-α,” “tumour microenvironment,” “immune checkpoint inhibitors,” “anti-inflammatory therapy,” “NF-κB,” “cancer therapies,” and “targeted therapies.” Boolean operators (AND/OR) were employed to enhance the search outcomes.

## Literature review

### Prevalence of cancer

As a major contributor to mortality globally, cancer plays a significant role, affecting one out of every nine people and accounting for around 12.6% of all fatalities globally Projections estimate a 70% rise in the annual incidence of new cases and fatalities between now and 2030 [8]. Cancer affects one out of every nine people and accounting for around 12.6% of all fatalities globally [8]. A study by [5] showed that cancer mortality rates are expected to exceed the worldwide mean by 30% in the upcoming twenty years. Infectious agents such as viruses, bacteria, and parasites have been associated with 25–30% of all cancer malignancies in Africa [8]. Moreover, in women, breast cancer is the leading cancer diagnosis followed by cervical cancer while among men, prostate cancer is the leading cancer diagnosis [9].

In South Africa, one of the leading causes of death is cancer accounting for about 10% of all national fatalities [9]. Based on research by [9], incident cancer cases are projected to increase twofold from 61 957 in 2019 to 120 969 in 2030. The cause for this growth is attributed to demographic and epidemiological changes as the South African population grows and ages. Projections from this study are that there is a 20% probability of having cancer before the age of 75 in South Africa, and a 10% chance of dying from it. The Western Cape Province has South Africa’s highest cancer death rate, followed by the Northern Cape and Limpopo Province and the cancer death rate in males and women is comparable. Moreover, cervical cancer is the most common kind of cancer in women, whereas lung cancer is the most common type of cancer in males in South Africa. Lung cancer, however, remains the largest cause of cancer mortality, followed by liver and stomach cancer [9]. Consequently, more investigations are needed especially with these three types of cancers and as a result this study will therefore be focusing on the liver cancer.

#### Liver cancer and its risk factors

Underlying liver diseases including hepatic injury and metabolic abnormities can lead to the development of liver cancer [10]. Primary liver cancer consists of hepatocellular carcinoma (HCC), cholangiocarcinoma (CCA), and other liver cancer types [11]. The absence of signs in the initial phases of the condition is often followed by a rapid progression of tumours, resulting in the majority of HCC cases being detected at an advanced stage [12]. Dietary exposure to aflatoxins, fungal poisons generated under high humidity, and temperature conditions for crop storage, is another key risk factor. These strong hepatocarcinogens contaminate dietary staples such as maize and peanuts result in a heightened possibility of liver cancer [4]. Excess body fatness, as well as behavioural and metabolic variables, can also be linked to liver cancer [8].

##### Role of hepatitis B (HBV) and hepatitis C (HCV) viral infections in liver cancer progression

According to [13] the biggest elements that increase the likelihood of liver cancer in Africa is Hepatitis B (HBV) and Hepatitis C (HCV) viral infections. Insufficient details regarding the origins of propagation, erroneous statistics regarding the condition’s impact, and low vaccination rate are the major factors contributing to the high rate of HBV in Africa. The Hepatitis B virus remains prevalent worldwide and is a significant contributor to the occurrence of HCC. HBV is transferred by interaction with the blood and bodily fluids of an infected individual because Hepatitis B has a direct carcinogenic effect on hepatocytes [14]. As HBV replicates, it has been demonstrated that it can integrate into the host’s DNA during the acute infection phase, resulting in oncogenesis through various mechanisms [15]. For instance, research has indicated that segments of HBV DNA can directly and indirectly stimulate gene activation and produce oncogenic proteins.

Prolonged HBV infection may trigger episodes of immune activation aimed at combating the virus, which can damage the liver and elevate the likelihood of tumor development. Elevated levels of ALT typically indicate these occurrences. Immune cells reacting to the virus have various subsequent effects: they secrete cytokines and chemokines that may promote cancer development, damage liver cells leading to quick cell proliferation and a heightened risk of mutagenesis, and assist in the generation of reactive oxygen species, which cause additional DNA damage [16]. Hepatitis C virus (HCV) can cause chronic liver disease, which can progress to fibrosis, cirrhosis, and HCC. During chronic HCV infection, chemokine-chemokine receptor interactions are particularly important for the recruitment of T cells to sites of inflammation in the liver. Carcinogenic features of the metabolic syndrome including uninhibited tumor growth, chronic inflammation, increased production of proinflammatory cytokines like c-Jun amino-terminal kinase 1 (JNK1), and reduction of anti-inflammatory proteins like adiponectin are mechanisms seen in NAFLD. NAFLD is a hepatic manifestation of the metabolic syndrome and considered a mechanism through which metabolic syndrome could lead to HCC[17].

##### Impact of hepatitis B (HBV) and hepatitis C (HCV) viral infections on chronic inflammation and liver cancer progression

Chronic inflammatory conditions accompany the advancement and evolution of these foundational liver conditions into cancer. Liver macrophages are recognized for their crucial functions in coordinating the inflammatory response [10].

As reported by the International Agency for Research on Cancer (IARC), liver cancer was the sixth most common type of cancer worldwide in 2020 and is the third major cause of deaths related to cancer. Furthermore, in 2020, it was estimated that there were more than 900,000 newly documented cases of liver cancer across the globe, resulting in approximately 830,000 deaths attributed to the disease. A key contributor linked to liver cancer progression is chronic inflammation, with up to 80% of cases being linked to chronic HBV and HCV infections. These infections trigger substantial inflammation in the liver, paving the way for the onset of liver cancer. The prevalence of long-term inflammation due to HBV and HCV underscores the substantial impact these viruses have on the global burden of liver cancer.

Liver cancer is a prevalent issue in Africa, being one of the most common cancers on the continent. According to the Global Cancer Observatory, 2020, liver cancer is the second most prevalent type of cancer among men and the fourth most prevalent among women in Africa. The annual incidence of liver cancer in Africa is alarming, nearly over 80,000 new cases were noted in 2020 alone. The mortality rate associated with this disease is also notably high, highlighting the urgent need for increased awareness, prevention, and treatment efforts in the region. In South Africa, liver cancer could potentially be less common compared to other forms of cancer, however it continues to be an important health issue. Furthermore, the South African National Cancer Registry (NCR) shows that liver cancer is among the top 10 cancers in terms of incidence in the country. The NCR data shows that every year, there are 2500 newly diagnosed cases of liver cancer in South Africa. Moreover, hepatitis B and C are identified as major contributors in the progression of liver cancer in the country. The prevalence of chronic hepatitis B is estimated to be around 4–5%, while chronic hepatitis C affects a smaller percentage of the population. Despite their lower prevalence rates, both hepatitis B and C are recognized as key contributors to liver cancer in South Africa. Given the global burden of this disease, particularly in areas where there are elevated occurrences of HBV and HCV; targeting chronic inflammation and its mediators represents a critical strategy in reducing liver cancer incidence and improving patient outcomes.

### Cellular and molecular mechanisms in liver cancer development

#### Importance of inflammation in liver cancer progression.

Persistent damage to the liver results in liver fibrosis and ultimately cirrhosis, which signifies a key stage in liver disease that can progress to HCC [4]. HCC is the leading kind of liver cancer worldwide, with cholangiocarcinoma coming in second [4]. An appropriate model of inflammation-induced malignancy is HCC since liver injury and persistent inflammation cause more than 90% of HCC cases. Additionally, viral, or non-viral infection-induced inflammation aids in the onset and development of HCC [18].

**Fig. 1:**
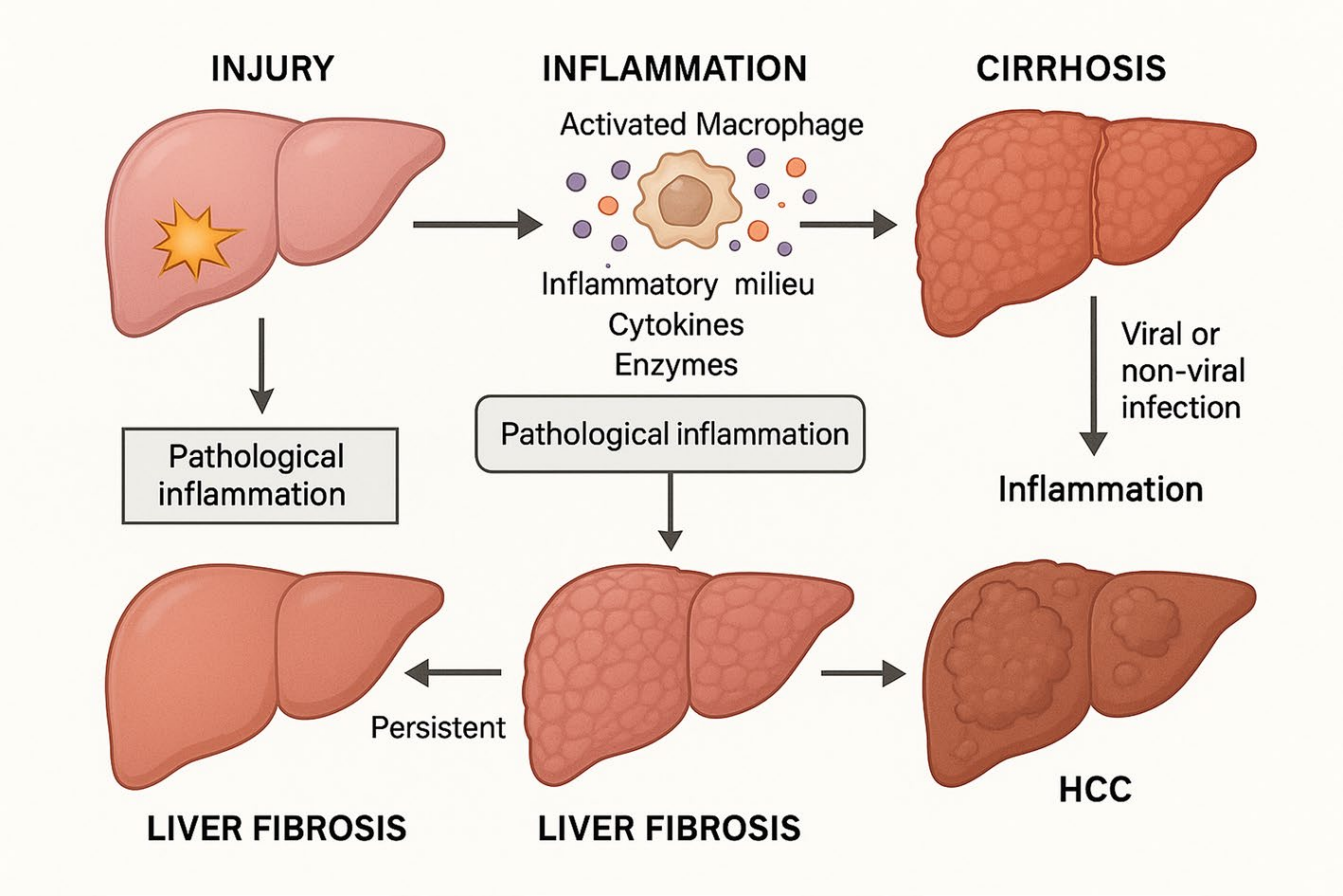
Inflammation in HCC progression

In typical circumstances, inflammation is a complex defensive response to injury or disease that comprises several processes aimed at preventing tissue damage [1]. However, as shown in Fig. 1, when inflammation is persistent and not adequately controlled, it leads to an undesirable condition known as pathological inflammation [1].

Inflammation triggers the activation of macrophages in the body resulting in a build-up of substances that promote inflammation such as cytokines, chemokines, and enzymes, which together create an inflammatory milieu [19].

##### Inflammatory cytokines

Inflammatory cytokines are proteins that cells generate when they encounter inflammation, triggering signalling pathways that activate the immune system [1] Chronic liver diseases, such as hepatitis infections, lead to prolonged inflammation which in turn triggers the production of pro-inflammatory cytokines that facilitate the growth and survival of cancer cells [20]. Various distinct cell populations produce both pro-inflammatory and anti-inflammatory cytokines [19].

Activated macrophages primarily produce pro-inflammatory cytokines, which are involved in regulating inflammatory responses [19]. Kupffer cells (KCs) and resident tissue macrophages produce pro-inflammatory cytokines when activated by phagocytosis or the binding of activation-inducing substances like endotoxins during HCC progression [21]. Cytokines such as interleukin-6 (IL-6), tumour necrosis factor-alpha (TNF-α), and interleukin-1 beta (IL-1β) [22] activate the downstream signal transmission pathway that includes the signal transducer and activator of transcription 3 (STAT3) which shifts the liver towards an oncogenic setting by promoting cell proliferation and inhibiting cell death [23].

The Janus kinase (JAK) family, which includes JAK1, JAK2, JAK3, and tyrosine kinase 2 (Tyk2), plays a critical role in the growth, survival, development, and differentiation of various cells, particularly immune cells. JAKs are vital for the intracellular signaling induced by cytokines in lymphocytes, and any dysfunction can lead to impaired immune cell function. Currently, it has been established that the clinical application of JAK inhibitors can enhance the treatment of several inflammation-related diseases. Research has shown that tofacitinib may be beneficial for autoimmune disorders by inhibiting the differentiation of harmful Th1 and Th17 cells, as well as affecting innate immune cell signaling. This indicates a potential for therapeutic use in liver diseases mediated by the immune system.

Furthermore, TNF-α stimulates the nuclear factor-κβ (NF-κβ) pathway, this pathway promotes liver inflammation and cell survival [24]. As a result, many chronic disorders linked with pathological inflammation have a larger uncontrolled production of pro-inflammatory cytokines like TNF-α and IL-6 [25]. In human liver cancer, research conducted by [26] indicated that various cytokines have been found to be elevated in the cancerous tissue of individuals diagnosed with liver cancer. The study showed that levels of IL-6 and TNF-α cytokines in patients with HCC were notably elevated compared to those in healthy human patients.

An animal liver cancer study conducted by [27] involving inducing liver cancer in rats, revealed that the concentrations of IL-6, TNF-α was increased in the liver tissue of rats with liver cancer compared to control group. This suggests that the inhibition of IL-6 and TNF-α could lead to reducing tumour growth and improving patient outcomes. Therefore, therapeutic interventions aimed at inhibiting IL-6 and TNF-α signalling pathways can potentially reduce inflammation and tumour proliferation. However, while their suppression may hinder cancer progression, excessively lowering these cytokine levels could impair the immune response, potentially leading to increased susceptibility to infections or other inflammatory diseases. Therefore, a balanced approach in cytokine targeting is essential, where therapeutic strategies aim to reduce pathological inflammation without compromising overall immune function. Furthermore, IL-6 is also involved in liver regeneration, particularly after injury. Suppressing IL-6 can therefore interfere with the way the liver manages to repair as well as regenerate.

Additionally, TNF-α plays a dual role in regulating inflammation and maintaining immune homeostasis. Its suppression can disrupt this balance, potentially leading to the development or exacerbation of autoimmune conditions [28].

**Fig. 2:**
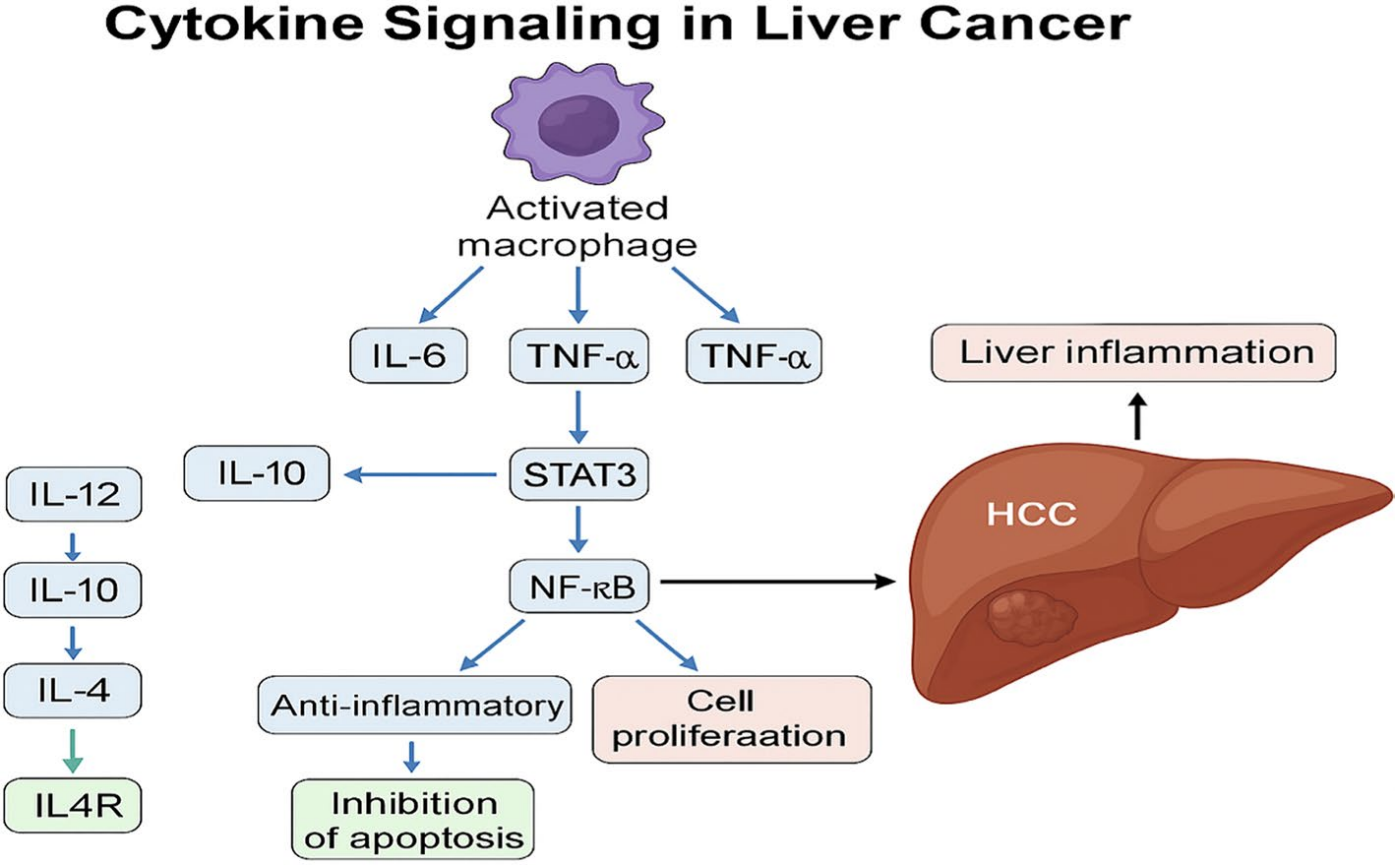
Cytokine signaling in liver cancer

Cytokines serve a key function in the immune response and have also been implicated at promoting and inhibiting tumorigenesis in liver cancer as shown in Fig. 2. Dysregulation of pro-inflammatory and anti-inflammatory cytokines can contribute to an altered immune environment, influencing cancer progression and patient outcomes. In liver cancer, specific cytokines such as Interleukin-12 (IL-12), Interleukin-10 (IL-10), and Interleukin-4 (IL-4) have been studied for their distinctive roles in modulating the tumour microenvironment. Interleukin-12 (IL-12) is a powerful pro-inflammatory cytokine, and in liver cancer it may be inhibited, leading to decreased immune responses against the tumour [29]. IL-10, on the other hand, is regarded as a key regulator of inflammatory processes that has been shown to possess a prophylactic effect in conditions influenced by pathological inflammation [19] IL-4 is another important cytokine, recognized for its multifunctional, pleiotropic nature. It is produced from activated T helper 2 (Th2) cells [30] and fulfils a crucial part during mediating as well as modulating immune and inflammatory responses. Additionally, IL-4 regulates cell proliferation, apoptosis, as well as the modulation of various genes in lymphocytes, macrophages, and fibroblasts. Recent research indicates that, beyond its role in immune functions, the interleukin-4 receptor (IL-4R) is often overexpressed among various cancers and may directly influence several signalling pathways involved in tumour development [30]. Notably, IL-4 and IL-4R have proven to protect HCC cells against apoptotic cell death triggered by transforming growth factor-beta [30]. Furthermore, IL-4R is expressed at significantly higher levels in cancerous tissues compared to normal tissues [30] Additionally, reducing IL-4R expression in HCC cells resulted in increased cell death, decreased growth, and lowered invasiveness of these cancer cells.

#### Reactive oxygen species

Reactive oxygen species (ROS) result from the metabolic processes involving molecular oxygen and are known as a mediator of inflammation because they induce pro-inflammatory cytokines [15]. TNF-α can stimulate the production of ROS which can cause deoxyribonucleic acid (DNA) damage at the same time, increasing the incidence of genomic DNA mutations [31]. ROS are produced are generated as a secondary product of cellular metabolism through the electron transport chain located in the mitochondria and cytochrome P450 [31]. ROS functions as both a signalling molecule and an inflammatory mediator, and at high levels or when not adequately controlled, they can cause cell damage and death [32].

Inflammatory cytokines can act on ROS and causes an increase of ROS levels in the body, this increase leads to oxidative stress that results in harm to nucleic acids, proteins, and lipids [33]. This damage may result in chromosomal instability and mutations resulting in cancer [14]. HCC is commonly linked to long-term infections caused by hepatitis viruses or the consumption of aflatoxins. Furthermore, these viruses can replicate and grow in human tissue [14]. Humans infected with these viruses stimulate the activation of phagocytes, which is linked to the generation of ROS. Additionally, the activated phagocytes can secrete proinflammatory cytokines [14].

#### How inflammation influences the tumour microenvironment in liver cancer

In liver cancer, inflammation is crucial in shaping the tumour microenvironment (TME), which is a diverse cellular environment composed of various types of cells, elements of the extracellular matrix, and signalling molecules [34]. Inflammation associated with carcinogenesis results in immune cells accumulating in the tumour and adjacent tissues, which can contribute to tissue remodelling and dysfunction [35]. The TME includes immune cells such as T lymphocytes, macrophages, neutrophils, dendritic cells; non-immune elements like fibroblasts, endothelial cells of blood, lymphatic vessels, and the extracellular matrix (ECM). These components create the environment that surrounds cancer cells. This microenvironment may have a dual function in tumour formation and advancement; it can support immune surveillance and immunoediting while also promoting tumour invasion, spread to distant organs, or evasion of immune detection [35].

Understanding the formation and advancement of HCC is essential for devising effective systemic therapies targeting HCC and for addressing resistance to existing treatments. The specific drug given and its efficacy will be influenced by the stage of cancer along with the liver cancer variant [35]. Prolonged inflammation results in the recruitment of immune cells into the liver, enhancing tissue remodelling. Various investigations into cancer biology demonstrate the interaction between cancer cells and the adjacent microenvironment significantly influences tumour advancement [36]. This interaction leads to the stimulation and enlistment immunocytes that possess immunosuppressive characteristics, ultimately hindering the body’s anti-cancer response. Consequently, cancer cells devise mechanisms to avoid detection by the immune system and promote the modification of immune cells to support tumour expansion. The establishment of this type of tumour microenvironment is essential for the initiation, advancement, and treatment response of HCC.

Additionally, the immune composition associated with the TME can differ from one individual with HCC to another. HCC has been categorized into four subclasses by [37] based on the composition of TME namely the; immune desert (C1), immunogenic (C2), innate immune (C3), and mesenchymal (C4). The C1 subclass is associated with a more favourable outcome and is devoid of primed T cells. In contrast, the C2 subclass is characterized by a low pathological stage and notable entry of macrophages, CD4 + . CD8 + T cells, and B cells. Meanwhile, C3 features initiated immunosuppressive M2 macrophages which is linked to poorest therapeutic outcomes. Furthermore, with regards to C4, activated cancer-associated fibroblasts (CAFs) facilitate the epithelial mesenchymal transition (EMT), which correlates towards the development of HCC [37, 38]

### Treatment of liver cancer

Primary treatments for hepatocellular carcinoma (HCC) include definitive procedures like partial or complete hepatectomy and liver transplantation. When surgery is not feasible, alternative treatments such as chemical, radiofrequency, or microwave ablation, and trans arterial chemoembolization are utilized [35], [39]. Liver transplantation and ablation may offer curative potential, but they are generally only effective for early-stage patients. Surgery is often restricted by factors like the size of the tumour and the quantity of nodules make it primarily appropriate for early detection [40]. Moreover, surgical resection is only suitable for individuals with a solitary nodule and satisfactory liver function, the situation is more favourable; however, those suffering from cirrhosis are at a higher potential for liver failure following surgery [41].

Numerous cases of HCC are detected at advanced periods because of the absence of particular tumour symptoms [35]. For these patients, systemic therapies are the primary approach, with sorafenib and other multi-kinase inhibitors now being the standard care for HCC. Conventional chemotherapies, often used for solid tumours, have been tested in HCC treatment without success [40]. For instance, doxorubicin showed no benefit in clinical trials when used with sorafenib, leading to increased toxicity without any enhancement in overall or progression-free survival compared to sorafenib alone [35]. Despite numerous efforts, treatment outcomes for HCC remain suboptimal, with frequent resistance to therapy. Moreover, the TME is believed to play a role in this resistance [42].

However, it is worth noting that In spite of these numerous attempts to discover effective HCC therapeutic strategies, the future health trajectory for patients continues to be low. The elevated death rate is mainly a result of late detection, which restricts possible treatment avenues, and the significant chance of recurrence following therapy. As a result, finding treatments that can prevent HCC recurrence is essential. Furthermore, since the TME significantly influences the progression of HCC and the resistance to standard treatments, immunotherapies are being explored as an additional strategy to reduce recurrence [43].

Interventional treatments, such as liver transplantation, are among the most effective options, addressing both HCC and underlying liver cirrhosis. This procedure is typically offered to individuals identified with early-stage HCC that is not resectable as well as leads to a 70% five-year survival rate. However, a major limitation is the shortage of available donor organs, resulting in many patients either dying while awaiting transplantation or becoming ineligible for the procedure.

Another invasive approach is trans arterial (chemo)embolization, a preoperative treatment and the primary therapy for individuals with mid-level phase. This approach entails obstructing the arterial blood supply to the tumour through the use of embolic agents, which may be paired with chemotherapy medicines. Its goal is to inject the chemotherapy straight towards the blood supply of the tumour while simultaneously cutting off its circulation, leading to tumour cell death through starvation [44]. Drug-eluting beads have been recently designed to decrease systemic toxicity while enhancing targeted delivery, offering similar effectiveness to standard trans arterial chemoembolization but with reduced side effects.

Radiofrequency ablation (RFA) and microwave ablation (MWA) are targeted therapies that use heat to induce cytotoxic effects to destroy tumour cells. The primary distinction between the two lies in their heat sources: RFA uses an electrical current in the radiofrequency range, while MWA relies on electromagnetic energy[45]. Both techniques are designed to target small, localized areas, with RFA effective in regions up to 2–3 cm in diameter and MWA treating areas up to 5–8 cm. These methods are typically employed in patients who are not eligible for surgery. RFA is particularly successful in tumours that are up to 2 cm in size, with a rate of survival of five years ranging between 40 and 70% [41].

A multi-kinase inhibitor known as Sorafenib targets VEGFR-1, VEGFR-2, VEGFR-3, platelet-derived growth factor receptor β (PDGFR-β), RET, c-KIT, and FMS-like tyrosine kinase-3. In the SHARP trial, sorafenib was shown to extend overall survival (OS) which improved from 7.9 months with a placebo to 10.7 months. Since 2007, it has established itself as the first-line therapy for advanced, unresectable HCC. However, treatment with sorafenib is associated with side effects, high costs, and resistance in nearly half of the patients. This resistance is thought to be due to certain immune cells associated with tumours [46, 47]. As a result, combining sorafenib with immunotherapy may help overcome this resistance. For almost ten years, sorafenib was the sole option for systemic treatment until the RESORCE trial, a global study that was randomized, double-blind, and placebo-controlled resulted in the approval of regorafenib in 2017. Regorafenib became the initial second-line therapy for individuals whose condition advanced following sorafenib. In the trial, regorafenib increased OS to 10.7 months for the treatment group, while the placebo group had an OS of 7.9 months, showing comparable efficacy to sorafenib [35].

The side effects that occurred most commonly consisted of skin reactions on the hands and feet, diarrhoea, fatigue, loss of appetite, high blood pressure, and oral mucositis. Adverse effects related to treatment occurred at double the rate within the regorafenib group in comparison to the placebo group [46]. Regorafenib targets a broader spectrum of kinases compared to sorafenib, affecting VEGFR-1, -2, -3, TIE2, PDGFR-β, FGFR1, KIT, RET, c-RAF, and BRAF [46], and demonstrated stronger anti-tumour effects in mouse models [46]. A year after regorafenib’s approval, Lenvatinib, a tyrosine kinase inhibitor that targets multiple receptors, was approved following the REFLECT trial [48]. Lenvatinib is also used as the initial treatment for inoperable HCC and is also the sole substitute to sorafenib that targets multiple kinases. It showed improvements over sorafenib in secondary outcomes, including increased objective response rate, prolonged progression-free survival, and an extended time to progression ([35].

Cabozantinib, another tyrosine kinase inhibitor targeting VEGFR-1, -2, -3, MET, and AXL, became the final kinase inhibitor to gain approval [49]. The CELESTIAL trial demonstrated longer OS in patients receiving cabozantinib which was 10.2 months, compared to 8 months for those on placebo, while progression-free survival was around 5 months for the cabozantinib group versus almost 2 months in the placebo group [35]. As a result, cabozantinib received FDA approval in 2019 as second-line therapy for patients who had previously undergone treatment with sorafenib (Abou-Alfa et al., 2018). Alternative kinase inhibitors, including sunitinib, linifanib, brivanib, tivantinib, and everolimus, did not demonstrate any life span advantages compared to sorafenib with liver toxicity and insufficient anti-tumour activity being the primary reasons for treatment failure [35].

Immunotherapies have become part of the treatment options for HCC, including the pairing of monoclonal antibodies atezolizumab and bevacizumab which targets PD-L1 and VEGF, respectively, Table 1 [50]. This combination received approval as a first-line treatment for individuals with unresectable, locally advanced, or metastatic HCC based on findings from the IMbrave150 study [35]

**Table 1:** Summary of immunotherapy for HCC – Atezolizumab + Bevacizumab combination

Therapy combination	Mechanism of action	Targeted molecules	Indication	Approval status	Key study
Atezolizumb + Bevacizumab	Monoclonal antibody (Immune checkpoint inhibitor) + Anti-VEGF antibody	PD – L1 (Atezolizumab) and VEGF (Bevacizumab)	Unresectable, locally advanced, or metastatic HCC	Approved as a first-line treatment option	IMbrave150 study

Furthermore, baseline serum IL-6 levels may influence the clinical response to this therapy, with lower IL-6 levels being associated with greater T-cell proliferation, cytokine production, and tumour-infiltrating T cells, leading to improved treatment outcomes [50].

## Discussion

The impact of inflammation on the advancement of liver cancer has garnered increasing attention in recent years, as it is now recognized as a crucial element in the progression and evolution of the illness. This discussion will explore the latest findings on how inflammatory mechanisms contribute to hepatocellular carcinoma (HCC) and examine advancements in therapeutic strategies targeting these pathways. Given that liver cancer remains a significant global health burden, understanding the inflammatory processes underlying its pathogenesis is crucial for developing more effective, targeted treatments. Several key findings have contributed to the scientific and clinical approaches aimed at modulating inflammation to control liver cancer progression.

### Inflammatory pathways and their targeting in liver cancer

**Fig. 3:**
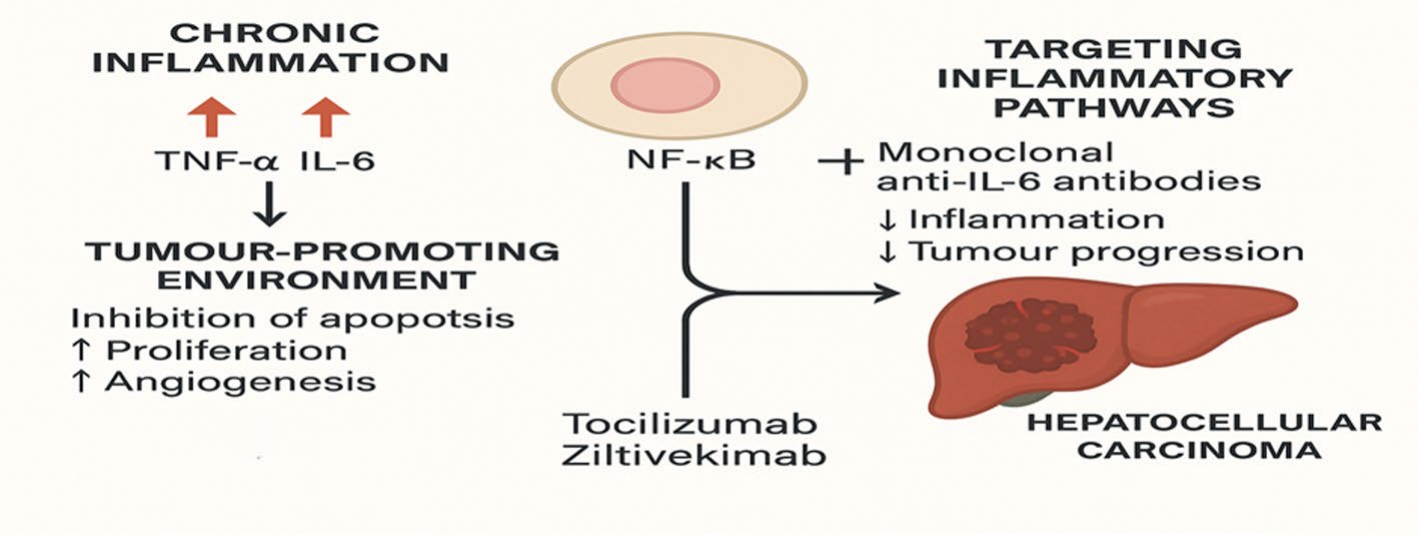
Cytokine signaling in liver cancer

Many studies as indicated in the preceeding sections have consistently demonstrated that chronic inflammation, primarily driven by cytokines such as TNF-α and IL-6, contributes significantly towards liver carcinogenesis, Fig. 3. These cytokines activate critical pathways, notably the nuclear factor-kappa B (NF-κB) signalling cascade, fosters a tumour-promoting environment by inhibiting apoptosis, enhancing cellular proliferation, and facilitating angiogenesis. Laboratory findings have highlighted that targeting these pathways, specifically by inhibiting TNF-α and IL-6, can reduce tumour growth and improve clinical outcomes in preclinical models of HCC.

Moreover, studies have successfully applied anti-inflammatory drugs and biological agents, such as monoclonal antibodies targeting IL-6, in clinical trials to modulate these inflammatory pathways [4]. Recent clinical trials have demonstrated promising results, particularly with agents like tocilizumab and ziltivekimab, which inhibit the IL-6 pathway. These drugs have shown efficacy in decreasing inflammation and potentially slowing tumour progression in HCC patients, especially those exhibiting high levels of inflammation-driven tumour growth [51]. Nevertheless, while these outcomes are encouraging, ongoing investigations are required to assess their safety and effectiveness over an extended period. More research is needed to assess how these therapies affect not only tumour size but also overall patient survival and quality of life.

### Challenges in anti-inflammatory treatments

Notwithstanding this progress, several challenges persist in targeting inflammation in liver cancer. Clinically, one of the primary difficulties is the delicate balance between reducing inflammation and maintaining immune function. While suppressing cytokines like IL-6 and TNF-α can be beneficial, over-suppression may lead to immunosuppression and increased susceptibility to infections, thus complicating treatment in cancer patients. Additionally, the heterogeneous nature of liver cancer complicates the standardization of treatments targeting inflammation, as patients may respond differently based on their tumour’s molecular profile and the extent of the inflammatory environment.

Furthermore, the complexity of NF-κB signalling presents a challenge, as this pathway can function both as a tumour promoter and a suppressor, depending on the context. This duality complicates therapeutic targeting, as over-inhibition of NF-κB could result in unintended consequences, such as enhancing tumour growth or compromising normal liver function [52].

### Emerging strategies: precision medicine and immunotherapies

Consequently, recent efforts have shifted toward combining anti-inflammatory therapies with precision medicine approaches to better tailor treatments to individual patient profiles. A promising avenue includes the combination of checkpoint inhibitors, including anti-PD-1 and anti-PD-L1 treatments which boost the immune response while controlling inflammatory pathways. Clinical trials have demonstrated that these agents, when used alongside cytokine inhibitors or anti-inflammatory drugs, offer a more targeted and balanced approach, reducing tumour burden while maintaining immune surveillance. Notably, studies such as the CheckMate 040 trial has demonstrated that Nivolumab, a therapy targeting PD-1, can result in long-lasting responses among patients with liver cancer.

Moreover, researchers have explored the potential of combining ICIs with therapies targeting cytokines like IL-6, which is heavily implicated in liver cancer progression. Preclinical and clinical studies have indicated that blocking IL-6, alongside PD-1/PD-L1 inhibitors, may offer a more effective treatment by mitigating the inflammatory environment that fuels tumour growth.

Furthermore, advancements in understanding the tumour microenvironment have allowed researchers to identify new targets, including stromal cells and immune cells that contribute to inflammation. Drugs that specifically target the pro-inflammatory components of the tumour microenvironment have shown efficacy in reducing tumour progression in preclinical models, laying the groundwork for potential clinical applications.

### Future directions and novel approaches

Looking forward, researchers have proposed several novel strategies to more effectively address inflammation in liver cancer. One emerging area of interest involves the use of nanotechnology to deliver anti-inflammatory drugs directly to liver tumours, minimizing systemic side effects and enhancing drug efficacy. Preliminary studies have shown that nanoparticles carrying TNF-α inhibitors or IL-6 blockers can localize treatment to the tumour site, thereby increasing the therapeutic window while reducing toxicity.

Another promising avenue involves modulating the gut microbiota to indirectly influence liver inflammation. Given that the gut-liver axis is vital in the processes of liver inflammation and carcinogenesis, scientists are investigating the treatment possibilities of probiotics, prebiotics, and faecal microbiota transplantation to reduce inflammation and slow cancer progression. Although this approach is still in its infancy, early data suggests it could complement traditional anti-inflammatory treatments.

Finally, gene-editing technologies such as CRISPR-Cas9 offer another promising approach to controlling inflammation in liver cancer. By specifically editing genes responsible for inflammatory cytokine production, researchers hope to develop more permanent and precise interventions for inflammation-driven cancer progression. While these techniques remain experimental, their high specificity holds great potential for future therapeutic applications.

### Limitations of the review

Although this review offers an overview of the current understanding of the inflammatory mechanisms related to HCC and new therapeutic approaches, it has certain shortcomings. First, the selection of literature was limited to articles published in English, which may have led to the omission of important information from non-English sources. Second, while reputable databases such as PubMed, ResearchGate, Google Scholar, and ScienceDirect were utilized, it is possible that some pertinent studies were overlooked due to publication bias or limitations in indexing. The review was also primarily centered on peer-reviewed articles, leaving out grey literature that might contain recent or initial findings. Finally, the fast-changing nature of research in this area indicates that new studies could have emerged following the review period, which was confined to publications from 2003 to 2024.

## Conclusion

In conclusion, significant progress has been made in understanding and targeting inflammation in liver cancer, with several promising strategies emerging from both laboratory and clinical research. While challenges remain in balancing immune suppression and inflammatory control. Advances in precision medicine, immunotherapies, and novel drug delivery systems offer hope for more effective treatments. Ongoing studies examining the impact of inflammation on the advancement of liver cancer, along with the creation of more anti-inflammatory strategies, will be crucial for advancing therapeutic outcomes for patients.

## Data Availability

No datasets were generated or analysed during the current study.

## Abbreviations

CCA: Cholangiocarcinoma
HCC: Hepatocellular carcinoma
HBV: Hepatis B Virus
HBC: Hepatis C virus
IARC: International Agency for Research on Cancer
NCR: National Cancer Registry
KCs: Kupffer cells
IL-6: Interleukin-6
IL-12: Interleukin-12
L-10: Interleukin-10
IL-4: Interleukin-4
TNF-α: Tumour necrosis factor-alpha
IL-1β: Interleukin-1 beta
STAT3: Signal transducer and activator of transcription 3
NF-κβ: Nuclear factor-κβ
Th2: T helper 2
ROS: Reactive oxygen species
PD-L1: Anti-programmed-death ligand-1
VEGF: Anti-vascular endothelial growth factor
VEGFR: Vascular endothelial growth factor receptor
PDGFR: Platelet-derived growth factor
TIE2: Angiopoietin 1 receptor
EGFR-2: Epidermal growth factor receptor 2
ECM: Extracellular matrix
TME: Tumour microenvironment
CAFs: Cancer-associated fibroblasts
B-RAF: B rapidly accelerated fibrosarcoma
FGFRs: Fibroblast growth receptors
